# Current Animal Model Systems for Ovarian Aging Research

**DOI:** 10.14336/AD.2021.1209

**Published:** 2022-07-11

**Authors:** Huan Lu, Lingwei Ma, Yan Zhang, Yanzhi Feng, Jinjin Zhang, Shixuan Wang

**Affiliations:** ^1^Department of Obstetrics and Gynecology, Tongji Hospital, Tongji Medical College, Huazhong University of Science and Technology, Wuhan, Hubei 430030, China.; ^2^National Clinical Research Center for Obstetrical and Gynecological Diseases, Wuhan, Hubei 430030, China.; ^3^Key Laboratory of Cancer Invasion and Metastasis, Ministry of Education, Wuhan, Hubei 430030, China.

**Keywords:** ovarian aging, model organisms, animal model systems

## Abstract

Ovarian aging leads to menopause, loss of fertility and other disorders in multiple organs, which brings great distress to women. For ethical reasons, it is impossible to use humans as direct study subjects for aging research. Therefore, biomedical researchers have employed different non-human organisms to study ovarian aging, including worms, fruit flies, fishes, amphibians, birds, mice, rats, cavies, rabbits, pigs, sheep, cows, horses, monkeys, and apes. Because each of these model organisms has its own features, multiple factors, such as size, anatomical structure, cost, ease of operation, fertility, generation time, lifespan, and gene heredity, should be carefully considered when selecting a model system to study ovarian aging. An appropriate model organism would help researchers explore the risk factors and elucidate molecular mechanisms underlying declined ovarian functions, which might be conducive to preventing or delaying the ovarian aging process. This article will offer an overview on several currently available and commonly used model organisms for ovarian aging research by comparing their pros and cons. In doing so, we hope to provide useful information for ovarian aging researchers.

Aging has become a hot research topic as human life expectancy increases, and a growing number of countries have entered an aging society. Aging is associated with a progressive loss of physiological integrity, impaired functions, and, ultimately, death [1]. Aging is highly associated with the development and progression of various human diseases, such as cancers, Alzheimer’s disease, osteoporosis, cardiovascular diseases, and cognitive dysfunction. Without treatment, aging-associated diseases are often lethal; thus, effective new therapies are urgently needed. Therefore, it is important to study aging in humans.

An early-onset aging phenomenon in humans is reproductive decline, which is particularly found in women, such as ovarian aging. Ovarian aging, the pacemaker of body aging [2], is a pathological process that is characterized by age-related gradual decline in ovarian function due to a decrease in quantity and quality of ovarian follicles, and is affected by many factors, such as genetics, environment, and lifestyle. It eventually manifests as menopause and affects multiple organs. Both natural and unnatural ovarian aging phenotypes lead to perimenopause syndrome, loss of fertility, and aging-related diseases. Damage due to ovarian aging occurs not only to the female reproductive system itself but also leads to poor oocyte quality and the declining health of the offspring, which could decrease the health level of the next generation. However, the mechanisms underlying ovarian aging are complex and remain incompletely understood. Primordial follicle activation and follicular atresia are two major biological processes [3]. Recent studies have indicated that gene mutations and DNA damage [4], mitochondrial dysfunction [5], increased apoptosis (especially oocyte and granulosa cell apoptosis) [6], decreased reductase activity [6], cellular senescence [7], aging of other organs (especially the hypothalamus) [8], neuroendocrine feedback [9,10], and many other mechanisms may be involved in the aging process of the ovary. Therefore, more intensive and systematic studies are needed to identify potential influencing factors and to decipher mechanisms for ovarian aging. Completing these studies will provide molecular basis and useful information for the development of a strategy for prevention of early or aberrant ovarian aging.

It is unethical and impractical to use humans as model systems. Hence, it is necessary to employ other non-human model organisms for ovarian aging research. Since Atria defined premature ovarian failure in the early 1950s [11], ovarian aging has been widely studied. A variety of organisms have been used as experimental models in this research because of their representative and facilitative characteristics. Among them, mice, rats, *Drosophila melanogaster*( *D. melanogaster*), and *Caenorhabditis elegans*( *C. elegans*) are the most commonly used model organisms [12]. The physiological properties of vertebrate model organisms, such as monkeys, dogs, rabbits, and mice, are similar to those of humans. Invertebrate model organisms, such as *C. elegans*and *D. melanogaster*, have been used for the study of ovarian aging, owing to their advantages of short lifecycles, high fertility, simple anatomical structures, and evolutionarily conserved genes. Studies using these model organisms have led to numerous ground-breaking discoveries and research advances, including the identification of new genes, protein functions, signaling pathways, pathophysiologic mechanisms, and potential therapies. The current research on ovarian aging is unsystematic, and the model organisms used by research teams may be different. Investigators should consider systematically selecting model organisms for ovarian aging research. A suitable model system would be helpful to uncovering influencing factors and molecular mechanisms of ovarian aging, as well as serves as a testing model for the exploration of effective prevention and intervention strategies.

In this article, we will offer an updated overview on currently available and commonly used model organisms for ovarian aging research by comparing pros and cons inFigure 1. As such, we hope to provide useful information for ovarian investigators.

## Invertebrates

### 1. Worms: *C. elegans*

The lifespan of *C. elegans* is 2-3 weeks at 20°C, and its generation time is 3-4 days (Table 1). *C. elegans*has five pairs of autosomes and one pair of sex chromosomes. The entire genome of *C. elegans* has been sequenced, and approximately 35% of its protein-coding genes share homology with human genes [13].

There are two types of *C. elegans*, male (XO) and hermaphrodite (XX). The hermaphrodite can be fertile by itself or inseminated by males, but male sperm are preferential. The hermaphroditic germline produces male gametes first, and then internal fertilization occurs and fertilized eggs are laid. The hermaphrodite lays approximately 300 eggs by self-fertilization and more than 1,000 eggs by allogamy. The whole reproductive system is distinct due to the transparent tissues of *C. elegans* [14]. The reproduction of *C. elegans* declines early and lasts one-third of its lifespan. Because of this feature, *C. elegans* has served as a model system for research on early decline in reproductive capacity. *C. elegans* has been exploited for apoptosis and senescence research. Molecular and genomic tools are further conducive to aging research by using *C. elegans* as a model [15]. By using the *C. elegans* model, researchers have found that a decline in the quality, not quantity, of oocytes causes reproductive aging [14]. Notch signaling is essential for germline aging [16]. One study [17] reported that 32 genes could be associated with delaying reproductive senescence, which provides new molecule insights into age-related reproductive aging. Apparently, *C. elegans* is also a suitable organism to identify new molecule players in regulation of ovarian aging and to elucidate molecular mechanisms underlying their roles in ovarian aging.

In summary, the advantages of *C. elegans* include a short lifecycle, high fertility, transparent tissues, genetic tractability, and low cost. It is a suited model for studies on the early decline in reproductive capacity, apoptosis, and senescence. The disadvantages are that *C. elegans* is a hermaphrodite and can be challenging to manipulate, so it is difficult to model human ovarian aging diseases using this organism.

### 2. Fruit flies: *D. melanogaster*

The lifespan of *D. melanogaster* is approximately 50 days at 25°C (Table 1). The development time is up to 8.5 days at 25°C, 7 days at 28°C, 11 days at 30°C, 19 days at 18°C, and >50 days at 12°C. *D. melanogaster* has four pairs of chromosomes, including an X/Y pair and three autosomes. A total of 139.5 million base pairs of the genome have been sequenced, and approximately 60% of *D. melanogaster* genes are conserved in humans.

Female *D. melanogaster* is able to copulate 8 h after hatching. Fertility peaks during the first week of adulthood [18]. At this stage, a female lays approximately 50-60 eggs per day [19]. The internal reproductive system of female *D. melanogaster* is composed of a pair of ovaries, oviducts, and a uterus [20]. The ovaries are shaped like a lotus in bud. There are three types of stem cell populations in the ovary, including germline stem cells (GSCs), somatic stem cells (SSCs), and escort stem cells (ESCs), which provide great insight into cell renewal and death [21]. Apoptosis, autophagic cell death, and necrosis occur in different cells in the ovary of *D. melanogaster* and have been associated with ovarian aging [22]. A variety of mutants and ovarian somatic cells have been identified. The use of CRISPR-Cas9 genome editing technology has enabled genome modification in ovarian somatic cells (OSCs) [23]. The UAS-GAL 4 system and RNAi are widely used in *D. melanogaster*. Currently, *D. melanogaster* is used for microRNA, small interfering RNA, and Piwi-interacting RNA studies [24].

**Table 1:** Model organisms and their features.

Species	Lifespan	Menopausal age	Reproductive life	Ovarian size	Ovarian weight	Estrous cycle	Gestation/ days	Singleton /Multiple pregnancy
**Human**	70-80 y	52 y	40 y	4×3×1 cm	5-6 g	28 days	280	1
**Apes**	40-52 y	37 y	30 y	-	-	30-33days	255	1
**Monkeys**	25-30 y	25-30 y	20 y	1.1×1×0.4 cm	0.4 g	23-33 days	156-180	1-3
**Pigs**	20 y	3 y	2.5 y	5×3×2 cm	7-9 g	18-23 days; all year	114	10
**Sheep**	5-15 y	-	-	1-1.5×0.5-1×0.5-1 cm	-	14-21 days; autumn and winter	150	2-4
**Horses**	30-60 y	25 y	20 y	7-8×3×3-4 cm	25-40 g	19-22 days; early spring-autumn	340	1
**Cows**	10-20 y	-	-	2-3×1-2.5×1-1.5 cm	15-20 g	20-21 days	275-285	1
**Rabbits**	5-12 y	-	-	-	-	8-15 days	28-36	7-12/birth; 5-6 births/year
**Mice**	1-3 y	-	-	0.2×0.1×0.05 cm	0.003 g	5 days; all year	19-21	4-8
**Rats**	2.5-3.5 y	-	-	-	-	4-5 days	21-23	10-12
**Cavies**	7-10 y	-	-	-	-	12-18 days; spring	59-72	1-6/birth; 5 births/year
**Birds**	2-80 y	-	-	-	-	-	2-7	2-300
**Amphibians**	~55 y	-	-	-	-	-	-	-
**Fish**	2-3 y	-	-	-	-	-	-	-
***Drosophila melanogaster***	50 d	-	-	NA	NA	NA	50-60 eggs/day	NA
***Caenorhabditis elegans***	2-3 w	-	-	NA	NA	NA	NA	NA

NA: Not available.

Because it was unclear what causes the decline in reproduction and by what mechanism this occurs during fly ovarian aging, it was proposed that in *D. melanogaster*, the decreased germline stem cell activity and increased incidence of cell death during oogenesis might be closely related to the age-related decline in reproduction [19]. Because of the possible role of senescence and imbalance of reproductive homeostasis in ovarian aging, antiaging strategies have been proposed and developed against senescence [25]. In addition, studies on the endocrine system in *D. melanogaster*provided a new discovery for dietary restriction to extend fly lifespan [26,27]. Finally, *D. melanogaster* has been used as a model system to screen for anti-ovarian aging compounds [28]. Therefore, *D. melanogaster*offers an effective and relatively inexpensive model system for ovarian aging research.

The advantages of a short lifecycle, high fertility, evolutionarily conserved genes, low cost, being dioecious, and having understood reproductive stem cells make *D. melanogaster* a great model for studies on apoptosis, autophagic cell death, and necrosis in ovarian aging. However, *D. melanogaster* die easily, and their small bodies are hard to operate on and to use to model the aging process of human ovaries.

**Figure 1.**
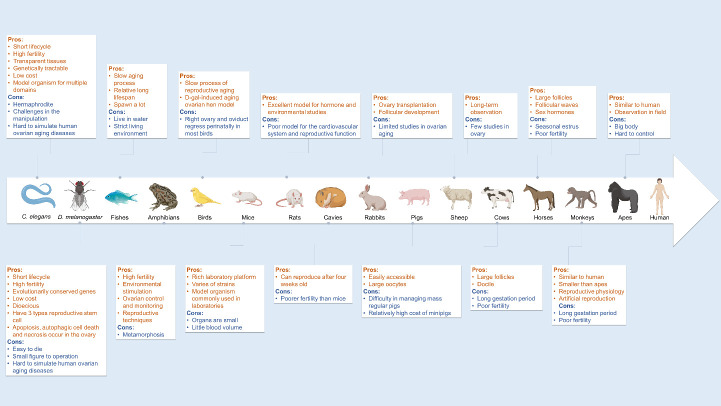
**Pros and cons of each model animal in ovarian aging research**. Created with from biorender.com.

Invertebrates have advantages of short lifecycle, high fertility, genetically tractable, low costs, small size, simple anatomy, and wide availability (Fig. 1). But they are always easy to die and hard to simulate human ovarian aging diseases.

## Vertebrates

### 1. Fish

The aging process of some fishes and reptiles is extremely slow [29] (Table 1). Fish spawn numbers ranging from hundreds to thousands or even hundreds of millions of eggs. The developmental process of fish ovaries can be generally divided into six periods according to standards for gonad volume, color, maturity of eggs, and so on. Only in phases IV2 and IV3, when the nucleus is eccentric-to-polarized, can artificial aphrodisiacs be successful (obtaining mature eggs). Some previous studies on the role of insulin-like growth factors in ovarian aging have been based on the *Takifugu rubripes* model [30,31]. The model fish *Oryzias latipes* has been used for some telomere and telomerase-related studies [32,33]. Zebrafish, which is small, inexpensive, highly fertile, and translucent, often serves as a popular genetic model system for studying ovarian aging [34,35].

### 2. Amphibians

Anura (frogs and toads), Caudata (salamanders), and Gymnophiona (caecilians) are three orders of Amphibia, and their reproduction can be categorized into seasonal and continuous breeders [36]. Amphibians have very different reproductive strategies based on the temperature and environment. Some females store sperm until they are ready to oviposit [37]. Amphibians are usually used for studies related to the impact of environmental stimulation on reproductive endocrinology and ovulation, as well as in research on ovarian monitoring and reproductive techniques [38]. Few studies on ovarian aging in amphibians have been reported.

### 3. Birds

The process of avian reproductive aging is gradual-to-slow senescence [29]. Birds live a long time and are suitable for developing models of ovarian aging [39,40]. Interestingly, although genetically remote from humans, the cohort of ovarian follicles and the cycle change of sex hormones in birds are similar to those in women. In most avian models, the right ovary and oviduct regress perinatally. The deposition of yolk and follicles is arranged in a hierarchy, similar to mammalian follicles. There are age-related changes in hypothalamic response, such as a diminishing luteinizing hormone (LH) surge and failing ovarian function in some birds, which is similar to women [12]. It is easy to observe the eggs and ovaries of hens. However, the morphometry and histology of the ovary in *Corvus macrorhynchos* change according to the season [41,42]. The D-gal-induced ovarian aging hen model has been established to investigate oxidative stress in ovarian aging [43,44]. In addition, studies of oxidative damage and reproductive senescence have also been conducted in short-life and rapidly aging bird models [39] (Fig. 1).

### 4. Mammals

#### 4.1 Rodents

Rodents have shorter reproductive cycles, including puberty, estrous cycle, pregnancy, and lactation, than larger animals. Their reproduction has been intensively investigated, as it is easy to give stimulation or intervention to rodents to study reproductive physiology and aging. Also, it is relatively easy for an investigator who studies ovarian aging to obtain any strain needed for ovarian research worldwide. Because of their relatively low cost, easy access, high adaptability, stable heredity, simple administration, clear structure, well-established disease models, and abundant databases, rodents are the most popular model organisms for research on ovarian aging as well as other diseases.

##### 4.1.1 Mice

Mice are the most commonly used mammalian animal model. Mice share the same organ systems as primates, including the cardiovascular system, digestive system, nervous system, and other systems, especially the urogenital system. Furthermore, mice are much less expensive and more effective in producing pups within a much shorter cycle than monkeys.

Indeed, *Mus musculus* is the most widely used laboratory animal in genetics, medicine, ethology, and other scientific disciplines. Ninety-nine percent of mouse genes are homologous to those of humans [45]. The lifespan of mice generally ranges from 1 to 3 years depending on the strain (Table 1). The commonly used laboratory strains are BALB/c, C57BL, ICR, KM, NIH, nude, and Scid. The internal reproductive system of female mice consists of two ovaries, two oviducts, a uterus, a cervix, and a vagina, similar to humans. However, two uterine horns or tubes are joined differently in a “Y” fashion. Breeding onset occurs at approximately 50 days of age in both male and female mice. They are polyestrous and breed all year around. Ovulation is spontaneous, and the pregnancy period is approximately 21 days. They can have 6-8 births per year with 5-10 pups per birth [46].

Laboratory mice can be used for modeling ovarian aging. Studies include exploring the mechanism of drug-caused damage to the ovary and determining protection strategies and methods. For example, ovarian aging models induced by gene deletion, chemotherapy, radiation, surgery, or environmental poisoning are commonly used for studying the mechanism of ovarian aging as well as screening for antiaging treatments.

Common chemotherapy drugs include cyclophosphamide, *cis*-platinum and paclitaxel. Cyclophosphamide and alkylating agents are usual chemotherapies for tumor patients, but these therapies will cause primordial follicle loss and oocyte death [47,48]. Therefore, it is crucial to develop an appropriate method to protect the fertility of cancer patients who receive chemotherapy. Using a mouse model system would be informative because the chemotherapy-induced ovarian damage model has been established using mice. Previous studies have shown that chemotherapy in mice leads to impaired estrous cyclicities and decreases in ovarian masses, follicle number, MII oocytes, and embryos, resulting in poor ovarian reserve, ovulation, and fertilization rates [3]. Remarkably, chemotherapy-induced ovarian damage has also been observed in women with diminished ovarian reserve and premature ovarian insufficiency (POI) [49,50]. Subsequent studies using the chemotherapy-induced ovarian damage mouse model system have shown that gonadotropin-releasing hormone (GnRH) and anti-Müllerian hormone (AMH) can prevent ovarian damage [51,52].

Radiation can damage ovarian follicles and accelerate ovarian aging [53]. Radiotherapy in mice has shown that the estrous cycle is disrupted, including ovarian weight, AMH levels, and follicle counts [54]. This mouse model is often used for drug tests. When irradiated mice were treated with tamoxifen, the drug was found to alter vaginal cytologic and ovarian histologic features during aging [55].

In addition, mice are widely used to study the effect of genetic alterations on ovarian aging. Chromosomal abnormalities and spontaneous genetic mutations account for the genetic causes of POI. Mouse models of POI are useful to decipher potential mechanisms. Genetically engineered mouse models, such as the follicle-stimulating hormone (FSH) receptor knockout (FORKO) mouse model [56], have shown the crucial role of FSH and AMH in oocyte development and survival [57]. The *Brca*2-deficient mouse model driven by *Gdf9-Cre* is infertile, demonstrating that *Brca2*, a core gene in DNA repair and homologous recombination, is required for follicle and ovary development [58]. Pigment epithelium-derived factor (PEDF) knockout mice were observed to have severe ovarian oxidative damage, which is related to the induction of severe insulin resistance and lipid metabolism disorder [59].

Ovarian aging leads to menopause in women, which can cause a series of perimenopausal diseases, such as osteoporosis and cardiovascular diseases. Ovariectomy (OVX) in mice simulates postmenopausal women whose estrogen level is often reduced markedly [60]. OVX-specific operations in mice are not complicated and can be performed in laboratories [61]. Moreover, OVX models are often used for Alzheimer’s disease [62], endocrinology, [63] and osteoporosis [64,65]. Bilateral OVX plus empty bottle stimulation offers a new way to easily establish a mouse model [66]. To evaluate the effect of cryopreserved ovary technology on ovarian aging, a mouse model system and a sheep model system were used to demonstrate that immature ovarian grafting could restore spontaneous puberty and fertility [67].

In addition, some basic experimental technologies have been applied in ovarian aging research. Vaginal cytology is used to evaluate the estrous cycle [68]. By using vaginal suppositories, it can be determined whether mating is successful. Measuring serum sex hormone levels and ovarian paraffin sectioning are used for the analyses of mouse ovarian functions and morphology.

Hence, mice provide the best model systems for ovarian aging research because of combined benefits including low-cost test subjects, rich strain resources, and the short lifespan of their mammalian reproduction system. However, there are some disadvantages of experimental mice, including limited volumes of ovaries and blood, making it hard to analyze these samples (Fig. 1).

##### 4.1.2 Rats

A rat has a lifespan of approximately 2.5-3.5 years (Table 1). The estrus cycle of female rats begins approximately 8-9 weeks after birth. The gestation period of rats is approximately 21-23 days, and a female rat can produce a litter of 10-12 babies [69]. SD rats can deliver as many as 20 babies. Remarkably, female rats can be estrous and pregnant again 12-24 h post-pup delivery. Rats have the same anatomies as mice. The most commonly used laboratory rat strains include SD, Wistar, Fisher344, SHR, and ACL.

Chemotherapy-induced premature ovarian failure (POF) models can be created in rats by intraperitoneal injection of cyclophosphamide [70,71]. In addition, POI rat models can be developed by oral administration of *Tripterygium wilfordii* polyglycoside [72] and pregnant mare serum gonadotropin (PMSG) followed by human chorionic gonadotropin [73]. A galactose-induced POI rat model has shown decreased fertility, ovarian weight, body weight, and follicle number, and increased atresia [74,75]. A 4-vinylcyclohexene diepoxide-induced model has been used to dissect the mechanisms of ovarian aging [76].

A polycystic ovary syndrome (PCOS) rat model is induced by administration of androgen or prenatal androgen to mimic menopause [77,78], and these studies have shown that hyperandrogenism-induced PCOS in rats is associated with ovarian aging-like phenotypes. PCOS and aging have similar symptoms, effectors, and commonalities [79]. The link between PCOS and aging needs additional investigation, but may be good for exploring the mechanism of ovarian aging. Additionally, postnatal androgenization leads to premature aging of rats [80]. Ovarian hyperstimulation syndrome in rats can be reproduced by injecting PMSG and human chorionic gonadotropin (hCG) [81].

When their ovaries were removed (100% left ovaries and 80% right ovaries) in OVX rats, the rats showed symptoms of menopause [82,83]. Meanwhile, rats displayed hot flushes associated with loss of reproductive function [84]. The pituitary-adrenal system of rats is well developed, and the pituitary is easy to remove. Rat models can be used for endocrine studies, such as the regulation of the hypothalamic-pituitary-ovarian (HPO) axis. Therefore, rats are convenient mammals to establish disease-model systems for ovarian aging research. But they are poor models for the cardiovascular system and reproductive function [84] (Fig. 1).

##### 4.1.3 Cavies

The cavy, *Cavia porcellus*, is also called the guinea pig. Guinea pigs can be pregnant year-round, with peak fertility usually concentrated in spring, and can deliver five litters per year. The gestation period is 59-72 days, with an average of 63-68 days (Table 1). A guinea pig can produce a litter of 1-6 babies, with an average of 3 and a maximum of 17. Female guinea pigs can reproduce after 4 weeks of age. Studies of follicle growth and pregnancy complications have been established in guinea pigs [85]. Although few studies on ovarian aging in guinea pigs have been reported and they need more living space than mice, guinea pigs serve as a good model system for ovarian aging research (Fig. 1).

#### 4.2 Lagomorpha

##### 4.2.1 Rabbits

Rabbits can live 5-12 years and reproduce after 6 months of age (Table 1). The estrous cycle of rabbits is 8-15 days. Rabbits are well-known for their high fertility. They can have 5-6 births per year and 7-12 babies per birth. A previous study investigated cell growth and programmed cell death or apoptosis in the corpora lutea of New Zealand White rabbits [86]. Young Lai et al. investigated the relationship between immunoreactive and biologically active FSH in the serum of sham-operated and OVX female rabbits [87]. Rabbits are suitable for autologous heterotopic ovary transplantation [88] and follicular development studies [89]. However, ovarian aging research using rabbit models is rare (Fig. 1).

#### 4.3 Artiodactyla

##### 4.3.1 Pigs

Pig fertility has been well described. The lifespan of pigs is approximately 20 years (Table 1). The internal reproductive system of a female pig consists of two ovaries, two oviducts, a bicornuate uterus, a cervix, and a vagina. The ovary is 5 × 3 × 2 cm in size and weighs 7-9 g. Breeding onset occurs at approximately 8 months of age in female pigs. They are polyestrous and breed all year around. The estrous cycle is approximately 18-23 days, usually 21 days. The pregnancy period is approximately 114 days. Pigs can have two births per year with 10 piglets per birth. After 6-7 births, their fertility begins to decrease.

Ovariectomized mature pigs are usually considered to be a good model for menopause-associated cardiovascular and bone studies [84]. Pig oocytes are used for *in vitro* cell research because their ovaries are easily accessible and larger than those of rodents [90,91]. Often, pig ovaries are taken after slaughter. Gene ontology analysis via high-throughput sequencing of ovaries from young and old pigs identified genes that encode proteins involved in histone and DNA methylation, and these genes have been shown to be involved in the ovarian aging cycle, including apoptosis, death effector domain binding, embryonic development, reproduction and fertilization processes, ovarian cumulus expansion, and the ovulation cycle [92]. New POI genes, *NRIP1, XPO1*, and *MACF1*, were identified to be linked to ovarian function in mice, pigs, and zebrafish [93]. Pigs are used for studies on cellular growth, natural aging, and programmed cell death [47]. Thus, pig model systems are appropriate for ovarian aging research (Fig. 1).

##### 4.3.2 Sheep

Sheep are another model system for ovarian aging research. Their lifespan is from 5 to 15 years (Table 1). Their ovaries are usually 1-1.5 × 0.5-1 × 0.5-1 cm in size. Sheep breed from autumn to winter, and the estrous cycle is 14-21 days. They produce 2-4 lamps per birth, and the pregnancy period is approximately 150 days. The ovaries of the sheep are located on the back of the kidney and are oval. Sheep ovaries are supported by the uterine ovarian ligament, bringing them close to the uterine horn.

Interestingly, the endocrine changes associated with increasing reproductive age in sheep are similar to those observed in women [94]. Sheep have a longer lifespan than rodents, which is propitious for long-term observation. Sheep are a promising large animal model for cardiovascular and bone systems [84]. Cryopreserved ovary technology [95] and ovariectomy in sheep have been confirmed to be effective for research using sheep as a model system [67,94,96] (Fig. 1). Other treatments include gonadotropin [97], dehydroepiandrosterone (DHEA) [96], and dihydrotestosterone (DHT) [98].

##### 4.3.3 Cows

The lifespan of cows is 10-20 years (Table 1). Cows have two ovaries, which are 2-3 cm long and weigh 15-20 g. Their estrous cycle is 20-21 days, and their gestation period is 275-285 days with a singleton pregnancy. One study compared 13-14 years old cows with their 1-4 years old daughters and concluded that aging is related to a delayed preovulatory LH surge after estradiol treatment. Older cows were prone to losing their large follicles after super-stimulatory treatment than were young cows [99]. Aging is associated with fewer 2-5-mm follicles at follicular wave emergence and a decreased follicular and ovulatory response after hyperstimulation treatment [100]. One group investigated the expression of HO-1 in bovine ovarian granulosa cells transiently exposed to heat stress and showed that heat stress induced oxidative stress and apoptosis and enhanced Nrf2 and HO-1 expression in primary granulosa cell cultures [101]. Other studies based on cow models include those of morphology [102] and dynamics [103] of ovarian follicles and endocrinology [104].

#### 4.4 Perissodactyla

##### 4.4.1 Horses

The lifespan of horses is generally 30-60 years (Table 1). Their ovaries are usually 7-8 × 3× 3-4 cm in size and weigh 25-40 g. Horses breed from early spring to autumn, until approximately 25 years of age, and their estrous cycle is 19-22 days. They have a singleton pregnancy, with a pregnancy period of approximately 340 days.

Compared with rodents or primates, mares and cows have far larger follicles, which provides greater feasibility for characterizing antral follicular dynamics by ultrasonography [105]. There are at least 12 similar characteristics between mares and women, especially in follicular recruitment waves [106] and the mechanism for selection of a dominant follicle [107]. However, the estrous period of a mare is in spring. Research on the role of sex hormones, such as AMH, FSH, and LH, in ovarian aging is usually based on the mare model [108-110]. For example, one group reported that AMH may reflect ovarian senescence in mares at 20 years of age [111]. Thus, mares serve as a good model system for ovarian aging research (Fig. 1).

#### 4.5 Nonhuman primates (NHPs)

Primatal model organisms are the closest model systems to humans, with similar anatomies, structures, physiology, pathologies, signs, symptoms, and pharmacological effects appropriate for clinical trials, including disease models and pharmacological experiments. The Nonhuman Primate Models of Menopause Workshop [112] was held to compare the menopause-associated changes in hormone levels during metabolism between rhesus macaques, baboons, cynomolgus, and chimpanzees [113-116].

##### 4.5.1 Monkeys

Monkeys are the most common primatal animal model. Among monkeys, macaques are commonly used for research in various areas. The anatomy of macaques is nearly the same as that of humans. They live up to 25-30 years (Table 1). Their bodies are smaller than those of humans and weigh between 5 and 8 kg. Females are sexually mature at 2.5-3 years of age and males at 4-5 years, but the first mating is at 6-7 years of age [117]. Their reproductive systems are similar to those of humans, and macaques aged 20-25 years undergo the menopausal transition, demonstrating evidence of ovarian senescence [118]. The uterus is 5 × 2 × 1 cm in size, and the ovary is 1.1 × 1 × 0.4 cm in size and weighs 0.4 g. Two ovaries are oblate and consist of albuginea ovarii, a medullary substance, and epithelium. Macaques menstruate from September to March or April, with a singleton pregnancy [119]. Interestingly, the principles of the endocrine control of women’s menstrual cycles were initially described in a macaque model [120]. There is a strong resemblance in reproductive endocrinology between humans and cynomolgus monkeys and some species of rhesus monkeys [121]. The age-specific gene expression profiles [122] and the proteome of adult rhesus monkey ovaries have been studied [6], and the genes associated with POF suggested that rhesus monkeys might be suitable for research on the mechanism of ovarian aging [123,124]. However, the histology of the ovary in neonatal marmosets displays an extremely immature phenotype [125]. Therefore, this animal model could be useful for the study of primitive gonad development and follicle assembly.

Macaques have also been used to investigate reproductive physiology, artificial reproduction, and family planning. Some common human diseases, such as vaginitis, cervicitis, endometritis, endometriosis, salpingitis, ovarian cysts, and ovarian tumors, have been observed in macaques [126]. These studies have led to some interesting findings. For instance, it was reported that humans are more prone to reproductive senescence than great apes [127]. Additionally, the analysis of the single-cell transcriptomic landscape of ovaries from cynomolgus monkeys indicated that oxidative damage is a crucial factor in ovarian functional decline with age [6].

Although primates are the perfect model to simulate human physiology, they are expensive to purchase and maintain for long-term research projects. Hence, they are suitable for the study of reproductive physiology, pharmacological experiments, and artificial reproduction[128] (Fig. 1).

##### 4.5.2 Apes

Apes include orangutans, gorillas, chimpanzees, bonobos, and gibbons. They are more similar to humans than are monkeys in physiology, but they are usually large, and they are hard to control in a laboratory. Apes live as long as 40-52 years (Table 1). They have a menopausal age of approximately 37 years, an estrous cycle of 30-33 days, and a gestation period of approximately 255 days, which varies among different types of apes. Like humans, they have a singleton pregnancy. One study compared the sexual behavior and regularity of progestogen cycles and found close concordance between hormonal cycling and sexual behavior in >40-year-old lowland gorilla females and <35-year-old gorilla females [129]

## Discussion and perspectives

The ovary, the core organ of the female reproductive system, secretes hormones that help maintain homeostasis and promote the function of multiple organs, including the brain, heart, and bones. The oocyte can give birth to the next generation. Hence, ovarian aging has great impacts on quality of life and health, as well as the health of offspring. It is very important to dissect the mechanisms underlying the occurrence and development of ovarian aging and to find strategies for delaying or preventing premature and/or pathological ovarian failure. However, ethical and practical limitations prevent scientists from using humans as a research model system. Therefore, choosing a suitable model organism is important for the study of ovarian aging. By describing various model organisms from the perspectives of complexity, characteristics, lifespan, reproductive lifespan, and ovarian cycle as above, we hope to provide useful information for ovarian aging researchers to pick up appropriate model systems for their constructing ovarian aging research platforms, unraveling novel ovarian aging mechanisms, and investigating innovative intervention strategies.

From *C. elegans* and *D. melanogaster*to rodents and primates, from inferior hermaphrodites to prior gonochorism, the ovary plays an important role in evolution, reproduction, and body health. A number of model organisms have been proven useful for the study of ovarian aging. The common advantages of small model organisms are small size, simple anatomy, low cost, easy operation, high fertility, short generation time, short lifespan, conservative gene heredity, and wide availability. Mammals, such as monkeys and apes, have similar anatomies to humans, so they are most appropriate for clinical trial experiments, such as pharmacology chemotherapy and other antiaging reproductive treatments. Primates can be used for artificial reproduction. Mares, pigs, sheep, and hens can be used for *in vitro* cellular experiments. *D. melanogaster* and *C. elegans* are appropriate for genetic, metabolic, environmental research, and preliminary drug screening. Investigators should choose different types of model organisms according to their research purpose so that their research goals can be efficiently and adequately achieved. The ultimate goal of research using model organisms of ovarian aging is to reveal molecular insights into the process of human ovarian aging. The use of these model organisms will help scientists better understand ovarian aging at the molecular, cellular, and genetic levels. Information gained from the studies will be advantageous for the development of possible therapies against ovarian aging and/or aging-associated diseases in the future.

## Limitations

The present review summarizes the commonly used model organisms. Additional model systems may include yeast or large and small organisms. As shown inTable 1, some data are unavailable. As there may be many different species in one genus, our table cannot include all the data. For example, the lifespans of birds vary from 2 years to 80 years; thus, a single number cannot represent all situations. The table and figure in this article summarize the assessable data, and more work is needed to generate further data.

There are many factors that influence ovarian aging. Investigators can choose suitable model organisms according to their interest of research. However, in addition to age-related factors, there are genetic, environmental, behavioral, and chemotherapy-related features. In the present review, we focused on age-related ovarian aging and did not include detailed information related to ovarian damage caused by other factors; however, it is necessary to investigate the mechanisms of, and interventions in, ovarian injury caused by such factors. Also, we did not include any model here for studying ovarian cancer that is clearly a crucial pathological factor for ovarian aging, too.
